# Toward the strengthening of radioprotection during mammography examinations through transparent glass screens: A benchmarking between experimental and Monte Carlo simulation studies

**DOI:** 10.3389/fpubh.2023.1171209

**Published:** 2023-03-31

**Authors:** Ghada ALMisned, Wiam Elshami, Elaf Rabaa, G. Kilic, E. Ilik, Duygu Sen Baykal, Antoaneta Ene, H. O. Tekin

**Affiliations:** ^1^Department of Physics, College of Science, Princess Nourah Bint Abdulrahman University, Riyadh, Saudi Arabia; ^2^Medical Diagnostic Imaging Department, College of Health Sciences, University of Sharjah, Sharjah, United Arab Emirates; ^3^Department of Physics, Faculty of Science, Eskisehir Osmangazi University, Eskisehir, Türkiye; ^4^Vocational School of Health Sciences, Medical Imaging Techniques, Istanbul Kent University, Istanbul, Türkiye; ^5^INPOLDE Research Center, Department of Chemistry, Physics and Environment, Faculty of Sciences and Environment, Dunarea de Jos University of Galati, Galati, Romania; ^6^Computer Engineering Department, Faculty of Engineering and Natural Sciences, Istinye University, Istanbul, Türkiye

**Keywords:** breast dosimetry, mammography, X-ray, radiation protection, MCNPX

## Abstract

**Introduction:**

A lead-acrylic protective screen is suggested to reduce radiation exposure to the unexposed breast during mammography. The presence of toxic lead in its structure may harm the tissues with which it comes in contact. This study aimed to design a CdO-rich quaternary tellurite glass screen (C40) and evaluate its efficiency compared to the Lead-Acrylic protective screen.

**Methods:**

A three-layer advanced heterogeneous breast phantom designed in MCNPX (version 2.7.0) general-purpose Monte Carlo code. Lead acrylic and C40 shielding screens were modeled in the MCNPX and installed between the right and left breast. The reliability of the absorption differences between the lead acrylic and C40 glass were assessed.

**Results and discussion:**

The results showed that C40 protective glass screen has much superior protection properties compared to the lead acrylic protective screen. The amount of total dose absorbed in the unexposed breast for C40 was found to be much less than that for lead-based acrylic. The protection provided by the C40 glass screen is 35–38% superior to that of the Lead-Acrylic screen. The C40 offer the opportunity to avoid the toxic Pb in the structure of Lead-Acrylic material and may be utilized for mammography to offer superior radioprotection to Lead-Acrylic and significantly lower the dose amount in the unexposed breast. It can be concluded that transparent glass screens may be utilized for radiation protection purposes in critical diagnostic radiology applications through mammography.

## 1. Introduction

Today, cancer and cancer treatment research are important topics of study for many medical experts (1). Due to its high prevalence and mortality rate among women, breast cancer has a prominent position among the many types of cancers (2). Scientific reports indicate that breast cancer is more manageable if detected at an earlier stage through breast screening programs (3). This is because the most significant tool for early diagnosis is breast screening (4), which is conducted using a variety of systematic methods, including mammography devices (4). Programs for breast cancer screening are a subject that is recognized and addressed in the health policies and public health programs of many countries around the world (5). It is also known that breast screening programs can minimize breast cancer-related mortality rates (6). Utilizing low-energy and ionizing X-rays and mammography equipment, which is important for breast cancer screening programs, is incredibly effective and practical (7). Mammography devices produce low-energy X-rays from the source and guide them onto the breast tissue under investigation. In accordance with the number of absorbed X-rays and X-rays that pass through the breast tissue and reach the image receptor, the patient's anatomic image is generated at the conclusion of the interaction process with the breast tissue. Glandular tissue and possible solid tumors and calcifications may produce X-ray absorption (8) due to their ability to absorb low-energy X-rays. In addition, they may be seen clearly on the collected radiological image as a result of the aforementioned X-ray interaction process (8). This procedure enables the detection of a likely risk factor and, subsequently, the early detection of breast cancer. On the other hand, the use of X-rays for diagnostic procedures should be considered alongside the advantages they bring and the potential negative consequences they represent (9). These risks include DNA mutations (10) generated by the impact of ionizing radiation on biological tissue and radiation-induced breast cancer formations (11). In other words, the mammography technique should be examined holistically, considering its advantages and the reduction in breast cancer mortality rate (12). Fortunately, researchers are extremely motivated to investigate and develop strategies and procedures that help mitigate these risks (13). Numerous international organizations and decision-making authorities, such as the ICRP, have already recommended specific action plans to mitigate these hazards (14, 15). The use of shielding materials and the removal or at least mitigation of unnecessary dose exposure are among the activities that may be performed (16, 17). It is widely known that several organs and tissues in the proximity of this region are also impacted by the dose emitted during breast cancer screening through mammography devices (18, 19). This dose scattering is an inevitable and natural result of the X-ray interaction with the biological tissue just like any material. In this circumstance, the most effective action to take is to prevent and minimize this dose's interaction with neighboring organs and tissues. In addition to the organs and tissues surrounding the exposed breast, the breast in the neighborhood of the exposed breast is also potentially exposed to these backscattered X-rays (18). Because each breast is irradiated two times in the craniocaudal (CC) and mediolateral oblique (MLO) positions during a routine mammography scan (20), the breast that is under examination is likely exposed to two separate exposures. Several effective investigations on the preservation of breast tissue outside of the extraction region have been conducted by researchers (21, 22). Among them, Koo and Lee (23) evaluated the dose received by the unexamined breast in the neighborhood of the exposed breast by positioning a lead-acrylic transparent protective screen between the two breasts. In their study, a substantial positive impact was found when the protective screen was utilized in terms of radiation protection. They have stressed that the implementation of the protective screen would considerably limit exposure to unnecessary doses. Although the transparency of the lead-acrylic material proposed by Koo and Lee (23) is a crucial factor for the management and monitoring of the other breast during exposure, the presence of toxic lead (24) in its structure may be harmful to the tissues with which it comes in contact. In this study, the protective properties of a cadmium-added glass screen, which may be used as an alternative to the lead-acrylic materials proposed by Koo and Lee (23), were examined and compared in terms of the same sizes and geometric properties. The acquired results might give crucial information for the development of protective screens, which can be an effective way of protecting radiosensitive organs and minimizing the prevalence of secondary malignancies caused by unnecessary exposure. In addition, given that glass materials are an important family of materials used in radiation protection (25) and that the literature has been intensively studied over the past few years (26–28), it is expected that the results would therefore make significant contributions to the literature of glasses that can be used for radiation protection.

## 2. Materials and methods

In the first phase, a basic phantom design was developed, which included the right and left breast, body phantom, and X-ray source. In the second phase of the investigation, standard data were used to evaluate the phantom's reliability. In the third and final phase, a separate design was constructed, which included the installation of a shielding screen between the right and left breasts. In this section, technical details about these three phases are presented.

### 2.1. Modeling and simulation details

**Figure 1:**
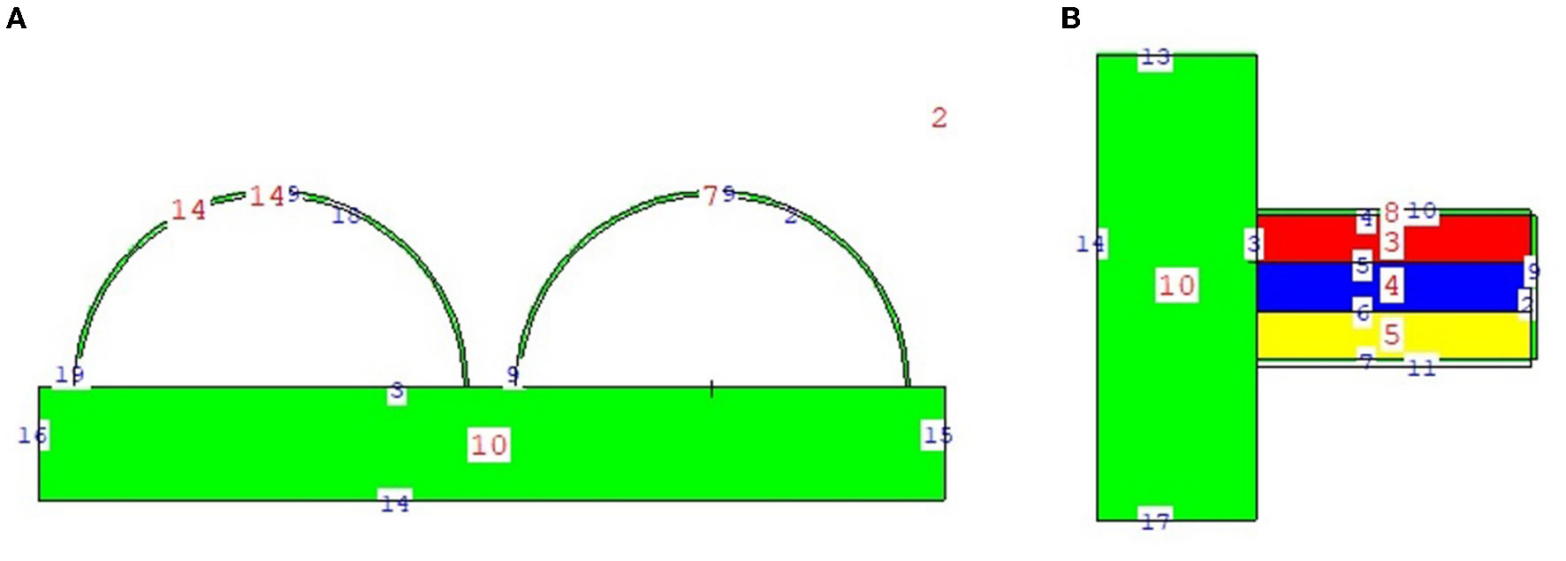
2-D view of modeled breast phantom from **(A)** top and **(B)** side view through MCNPX visual Editor.

**Figure 2:**
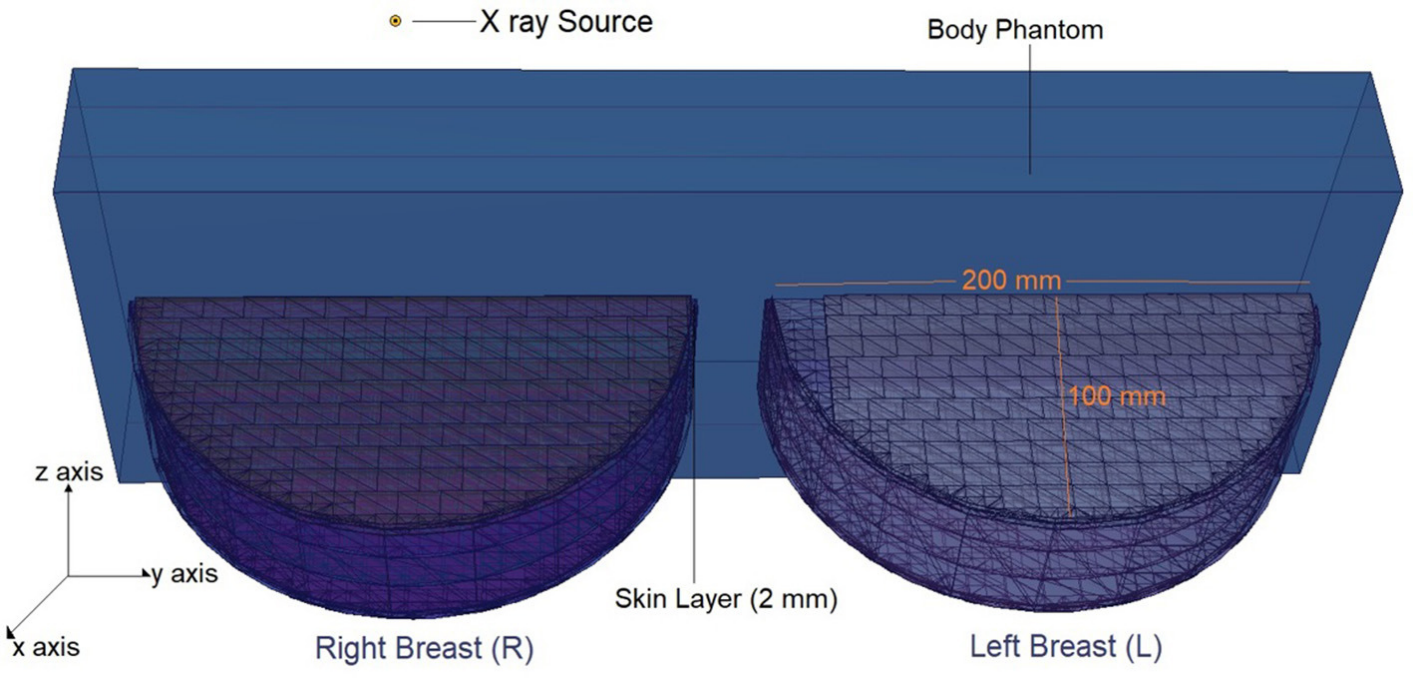
3-D view of modeled breast phantom along with dimensions and simulation axis from top view through MCNPX visual Editor.

**Figure 3:**
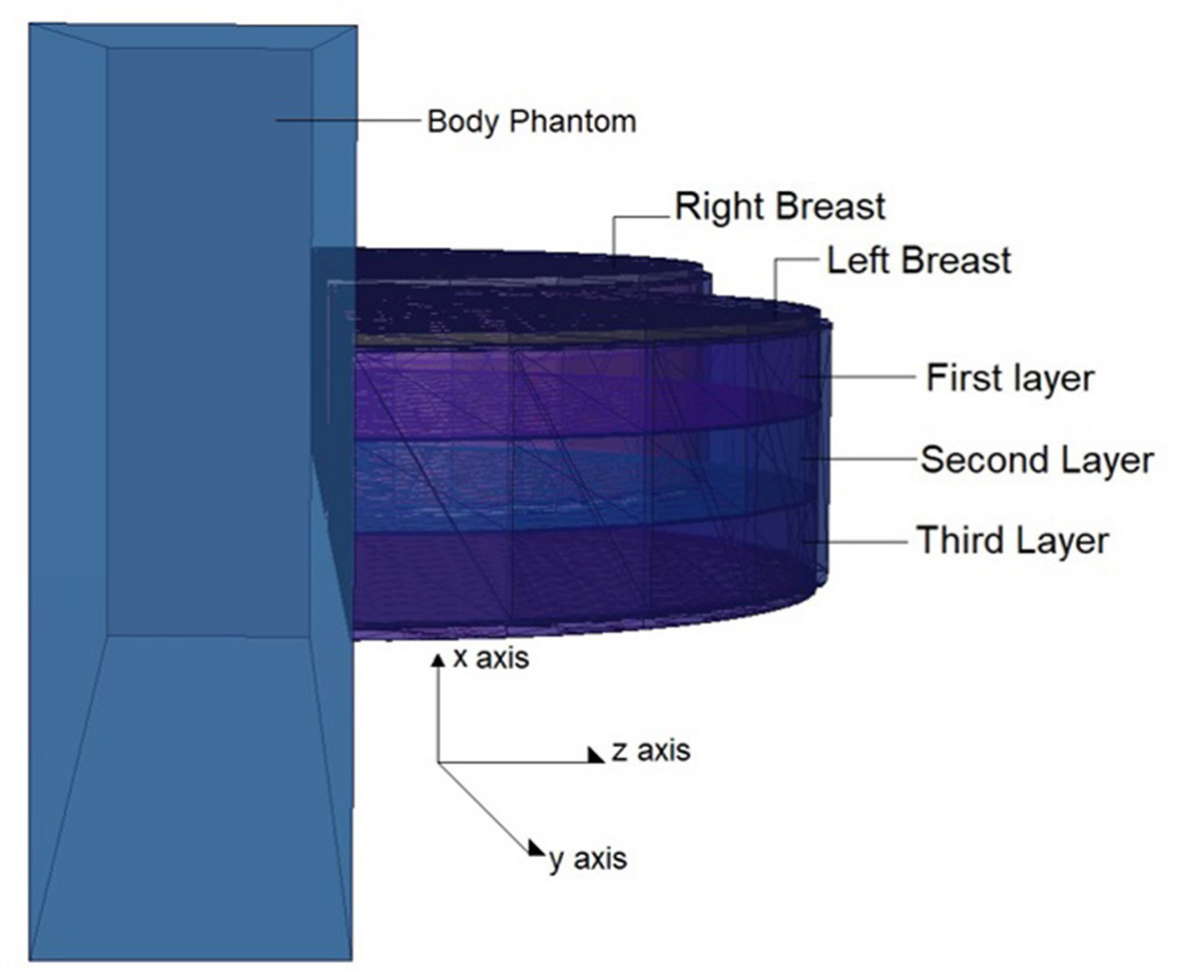
3-D view of modeled breast phantom along with the positions of breast layers from side view through MCNPX visual Editor.

The physical construction and all modeling steps of the breast phantom used in the investigation were accomplished using version 2.7.0 of MCNPX (29), a well-known and general-purpose Monte Carlo tool. Eventually, a breast phantom with three layers was generated. A unique Glandular Fraction value was given to each layer in the left and right breasts. This three-layer heterogeneous breast model has recently been proposed by Chang et al. (30) for advanced breast dosimetry studies. Figure 1 shows the 2D view of modeled breasts along with the body phantom from (a) the top and (b) the side view. The glandular fraction (GF) coefficients of the modeled layers were derived in line with a previously studied geometric principle. Each layer was designated as a unique CELL inside the MCNPX input file. The contents of these CELL volumes, as well as their elemental percentage fractions and densities, were provided in the INPUT file in accordance with the different GF values. In addition to their constituent percentages and densities (see Table 1), the skin layers around the five surfaces of the three-layer breast model were also specified for the left and right breasts. On the back of the generated breast model, a human-tissue-dense body phantom was incorporated toward the input. In the final stage, a breast phantom-specific source with a source-image receptor distance of 65 cm was designed. Figures 2, 3 show the 3D view of the modeled breasts from the top and the side view. Figure 2 demonstrates that a 2-mm skin layer was placed on both breasts. Moreover, the X-ray source was placed over the right breast. The first is the primary layer with which the primary X-rays will interact based on the direction of their entrance. X-rays that pass through the first layer will correspondingly penetrate the second and third levels. These layers' absorption will decrease the number of X-rays that reach the image detector. Figure 3 depicts, from the side, the sequence of layers in the modeled breast phantom based on the location of the X-ray source.

**Table 1:** Elemental properties and densities of the modeled breast layers (30).

		**Weight percentage (%)**			
**Tissue**	**Density (g/cm** ^3^ **)**	**H**	**C**	**N**	**O**
GF tissue (25%)	0.955	11	51	2.1	35.7
GF tissue (50%)	0.982	10.7	40.1	2.5	46.4
GF tissue (75%)	1.010	10.5	29.3	2.9	57
Skin	1.090	9.8	17.8	5	66.7

### 2.2. Validation of MCNPX

In the first step of the investigation, the MCNPX code verification procedure was conducted. This verification process was conducted in accordance with the dosimetry guideline for mammographic techniques detailed in the AAPM TG-195 (31) report. Using the report's specified parameters and dimensions, a breast phantom with an adipose-to-glandular tissue ratio of 80 to 20% was modeled. More technical information on the breast phantom model may be found in the resource handbook (31). Using the F6 Tally Mesh, a major tally variation of the MCNPX algorithm, the quantity of energy deposited per unit volume is then computed. Based on three iterations, this procedure yielded a result of 4,782 eV/photon (4,782 eV energy transferring per photon). Compared to the data in the source guide, a discrepancy of 3% was found. This variance is within acceptable limits, and it is believed that it is attributable to computer hardware, processor, and other simulation features. Once verification was accomplished, the next steps of the investigation were carried out.

### 2.3. Modeling of lead-acrylic and C40 shielding screen

**Figure 4:**
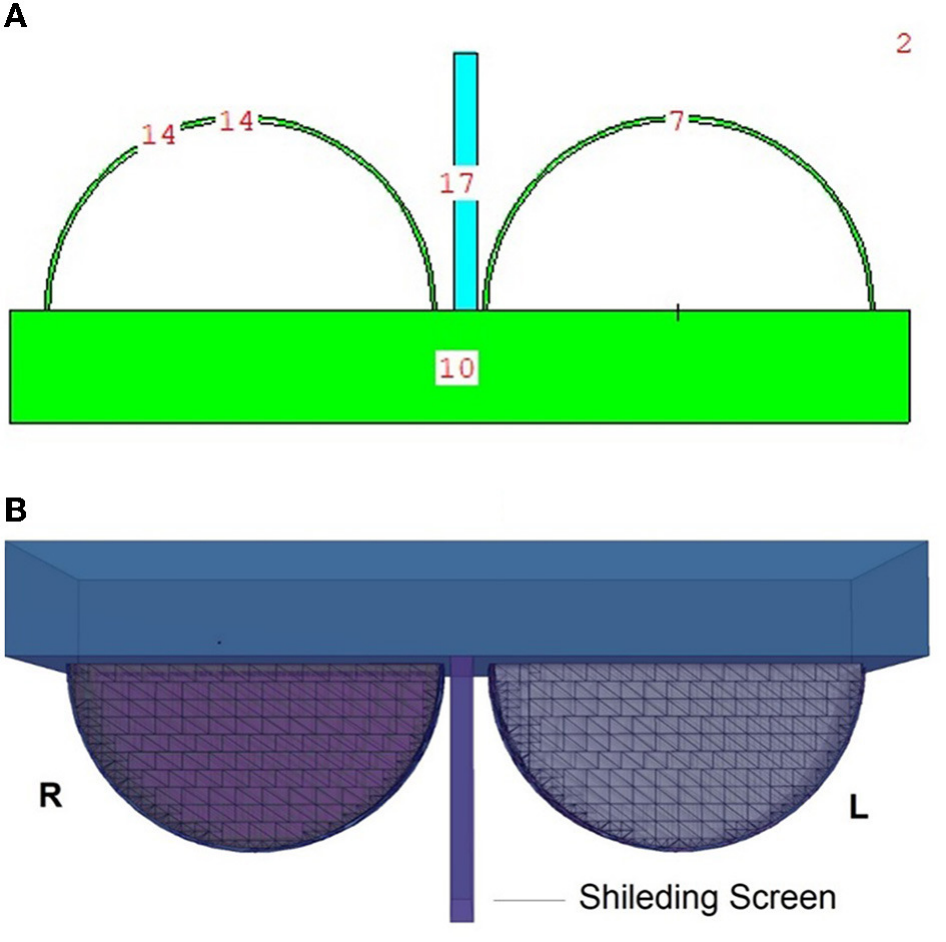
**(A, B)** Depict the 2D and 3D top views of the shielding screen positioned in the geometric center of the breast phantom model, respectively.

Following the basic modeling outlined earlier, a second input code file was created. The difference in this second input code was the shielding screen that was placed between the right and left breasts, and the input file included both surface and cellular definitions of the modeled screen using elemental mass fractions (wt.%) and material density (g/cm^3^). The dimensions of the modeled shielding screen are defined as 20 × 20 cm^2^. In later phases of the study, the structure of the material in the input file was defined separately as lead-acrylic (23) and C40 cadmium oxide-rich glass. This was done to analyze the absorption differences between the lead-acrylic and C40 glass (32) screens, which was also the primary purpose of the study, and to determine how much it decreased the dose delivered to the unexposed breast. It is worth mentioning that the C40 glass screen was synthesized by the research team (32) as a CdO (cadmium oxide)-rich quaternary tellurite glass for nuclear safety purposes. In a previous study (23), it was found that the absorption equivalent of a 12-mm thick lead-acrylic material is comparable to that of a 0.5-mm thick lead (Pb). In the reference study (23), lead acrylic is recommended as a shielding screen with dimensions of 20 × 20 cm^2^ and a thickness of 12 mm. Consequently, the proposed lead acrylic with the same dimensions was modeled in the MCNPX and inserted between the right and left breasts. Next, the same dimensions were used for the design of the C40 shielding screen as well. Using the specific properties, such as elemental mass fractions (wt. %) and density (g/cm^3^), for C40 glass, the cellular structure line was modified in the input file, yet the geometric dimensions in the surface cards remained unchanged. This enabled us to examine the absorption properties of C40 transparent glass, which has the same dimensions and thickness as the specified lead acrylic, with dimensions of 20 × 20 cm^2^ and a thickness of 12 mm. The center of the shielding screen was determined as the geometric center of the left and right breasts. Figure 4 depicts the 2D and 3D top views of the shielding screen positioned in the geometric center of the breast phantom model. Meanwhile, the C40 sample, which is rich in cadmium oxide, was manufactured by our team and was suggested in previous studies as a promising material in terms of its transparency and radiation-absorbing properties. The primary objective of this research was to investigate the applicability of C40 material for medical procedures, considering its transparency and exceptional radiation absorption qualities.

## 3. Results and discussion

**Figure 5:**
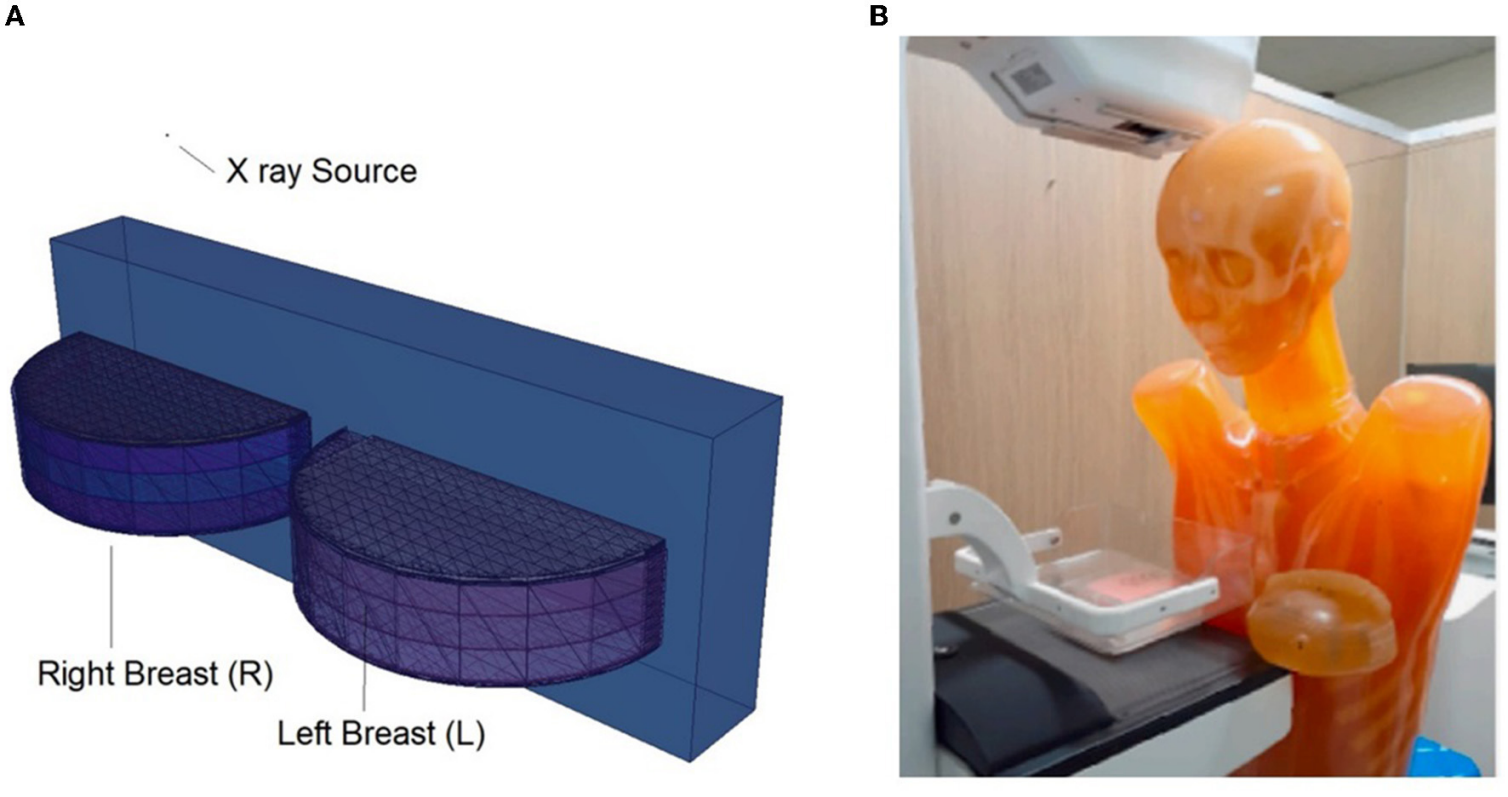
**(A)** Modeled left and right breasts. **(B)** Physical phantom and its positioning (23).

**Figure 6:**
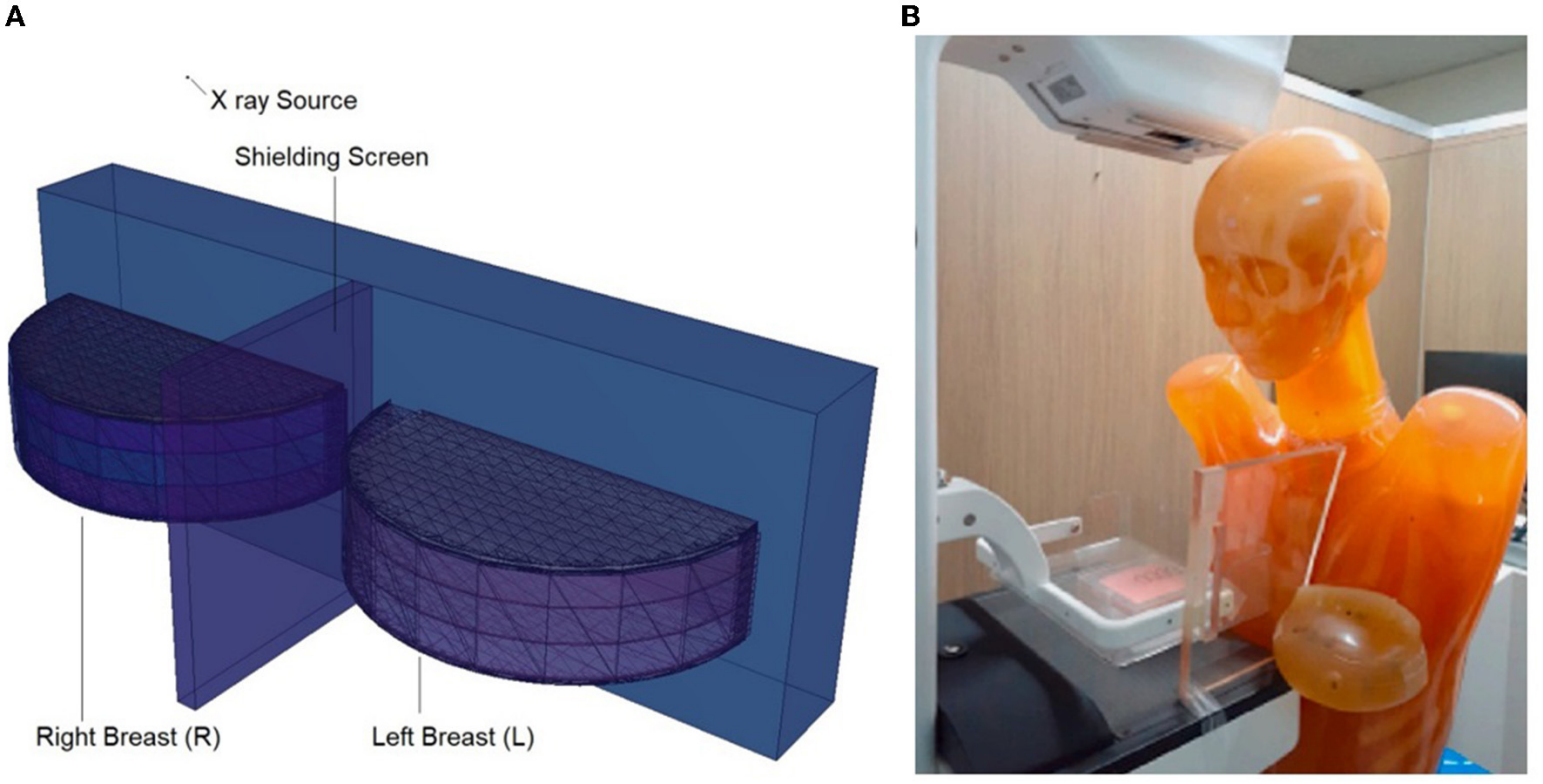
**(A)** Modeled breasts along with the shielding screen. **(B)** Physical phantom and positioned Lead-Acrylic screen (23).

**Figure 7:**
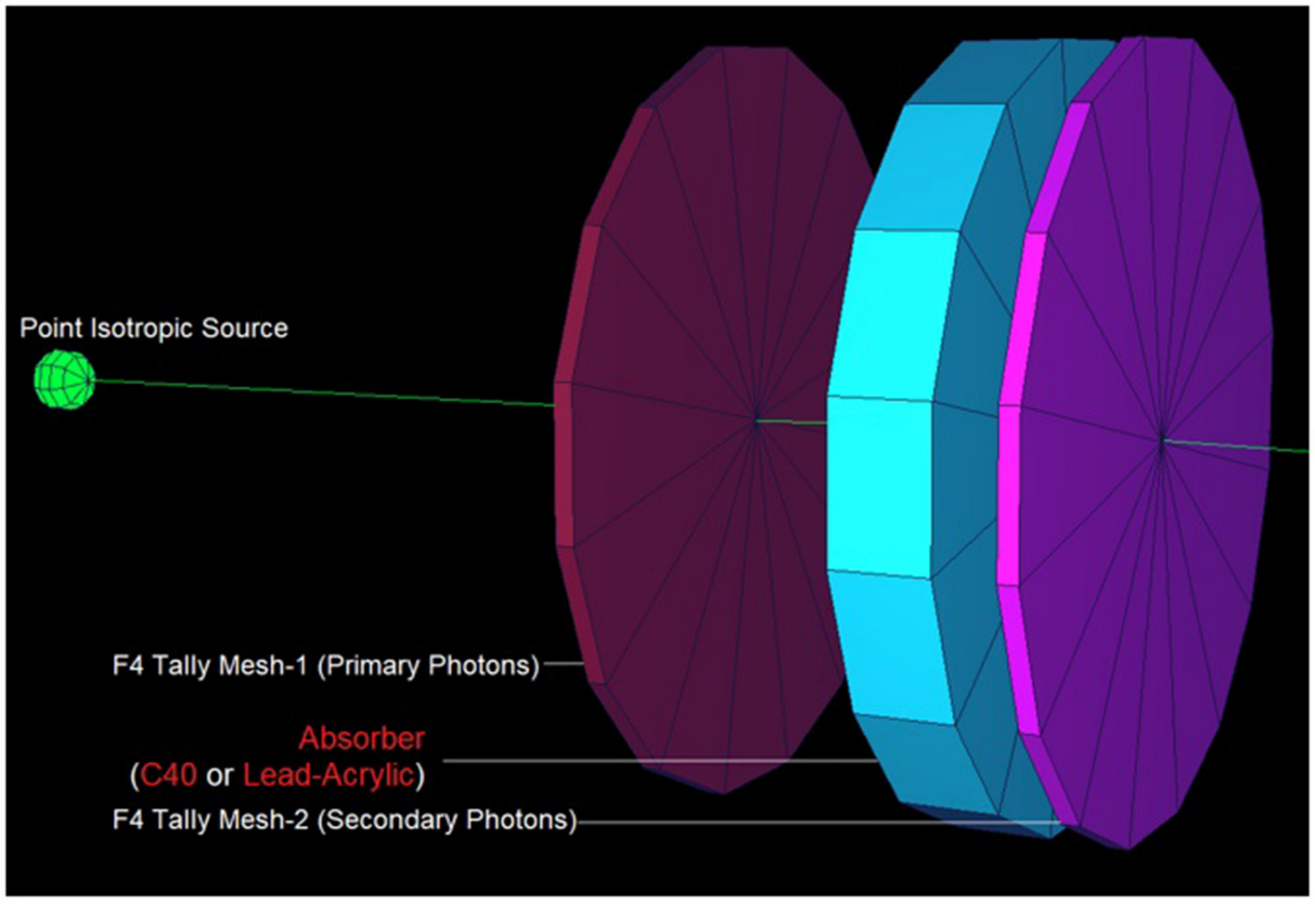
Designed MCNPX simulation setup for TF calculations for C40 and Lead-Acrylic protection screens.

In this study, the reduction in the amount of dose absorbed in the non-exposed breast compared to the exposed breast caused by the mammography device used in routine breast screening procedures was investigated. A fully-fledged breast phantom design, as indicated by international publications, was developed for the investigation. In a previous study published (23) in the scientific literature, the dose to the non-exposed breast was lowered using a lead-acrylic protection screen, and substantial results were observed. In this study, the experimental phantom was designed with all its geometrical characteristics and positioning, and the suggested protective C40 transparent glass screen for the unexposed left breast was thoroughly compared with the experimental data obtained through a lead-acrylic protection screen. In the first phase of the study's results, the experimental phantom shown in Figure 5B was designed in MCNPX. Although a uniform breast phantom was employed in the experimental investigation, a three-layer advanced heterogeneous breast phantom (see Figure 5A) was used in this study to raise the degree of coverage of the results for each modeled breast layer. This allowed the researchers to study the protective characteristics of the protective screen on a layer-by-layer basis and make more specific recommendations as a function of the modeled breast layers from the first to third breast layers, respectively. Figure 6B depicts the positioning of the lead-acrylic protective screen utilized in the experimental investigation between the two breasts, as well as the condition of the left breast and the positioning of the lead-acrylic protective screen during exposure to the right breast. This experimental setup was constructed similarly in MCNPX Monte Carlo code seen in Figure 6A, and the protective screen was positioned between two breast structures with equal experimental dimensions. Meanwhile, prior to breast dosimetry investigations, the transmission factors (TF) of the compared protective screens were determined. TF is the mathematical ratio between the intensity of the initial photon encountered on one side of a material and the intensity remaining on the other side of the material. These values are physical characteristics that provide crucial information about the material's absorption capacity, and they are recognized as an absorption parameter utilized in numerous investigations. Prior to examining the radiation shielding properties provided by the two compared shielding materials, the primary objective of this pilot study was to obtain some different comparison results that would validate the absorption properties to be obtained in the subsequent phases by observing the percentage reductions in the photon intensity at specific energies. For this initial phase, two detection zones in front of and behind the absorber material were constructed. As the main gamma-ray intensity, photons generated by an isotropic point source were evaluated. After interacting with the absorber material, photons that were counted as the secondary photon amount on the material's backside were also tallied in the detection region behind the absorber material. Figure 7 depicts the TF values and the rate of change of these values from a lead-acrylic screen to a C40 glass screen by calculating the ratios of the secondary photon quantity to the primary photon quantity. As demonstrated in Figure 7, the number of primary photons was seen to be the same in both situations. This is an anticipated result of using the same energy value as the source. However, the values of secondary photons were different, with a lower value for C40. This decrease in the number of secondary photons observed for C40 was reflected in the proportionality value resulting in a lower TF for C40 (see Equation 1).


(1) $$\begin{array}{l} \begin{array}{l} \text{Transmission Factor }(\text{TF})=\text{Photon Flu}X_{\text{Secondary}}/ \end{array}\\ \;\begin{array}{l} \text{Photon Flu}X_{\text{Primary}} \end{array} \end{array}$$

**Figure 8:**
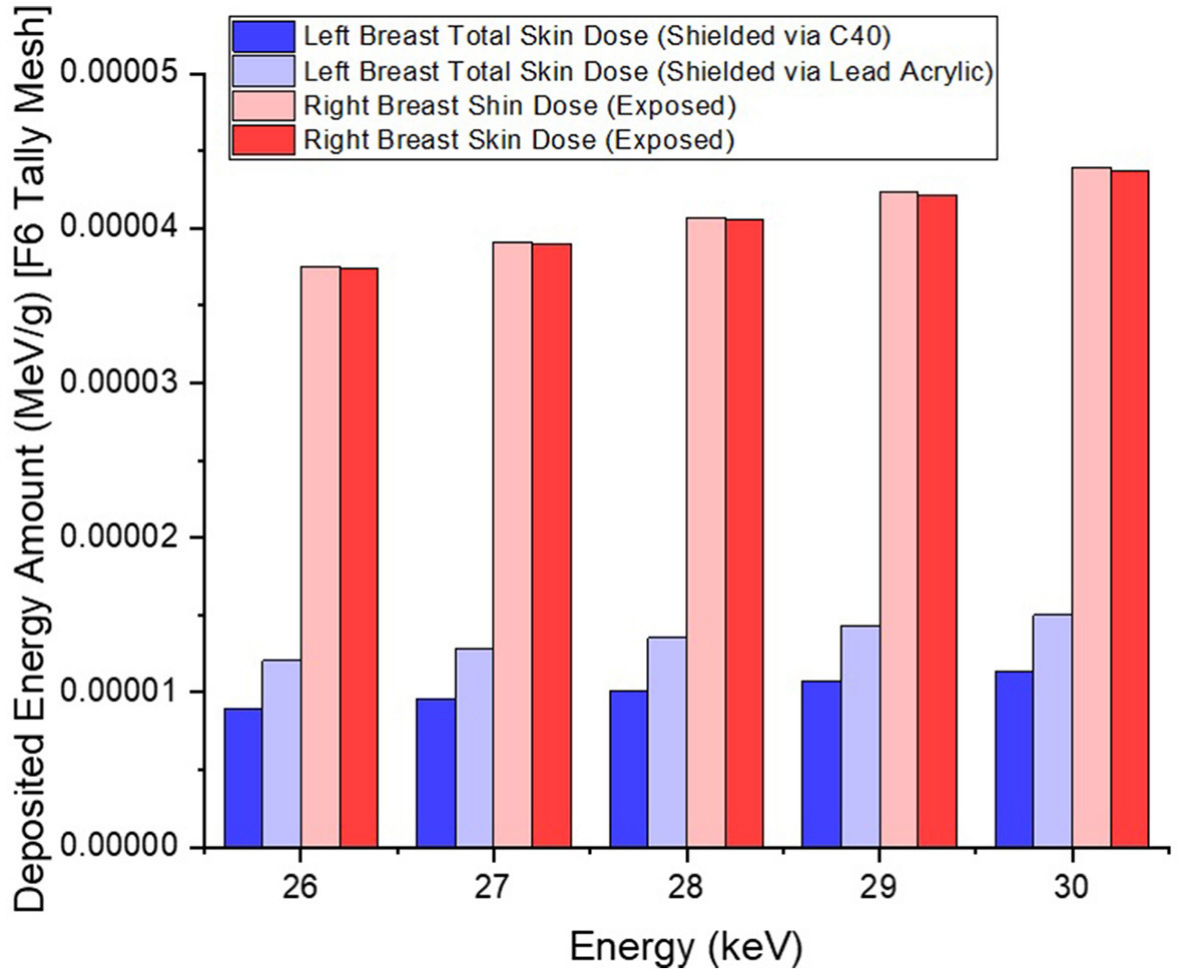
Variation of total skin doses for left and right breasts.

**Figure 9:**
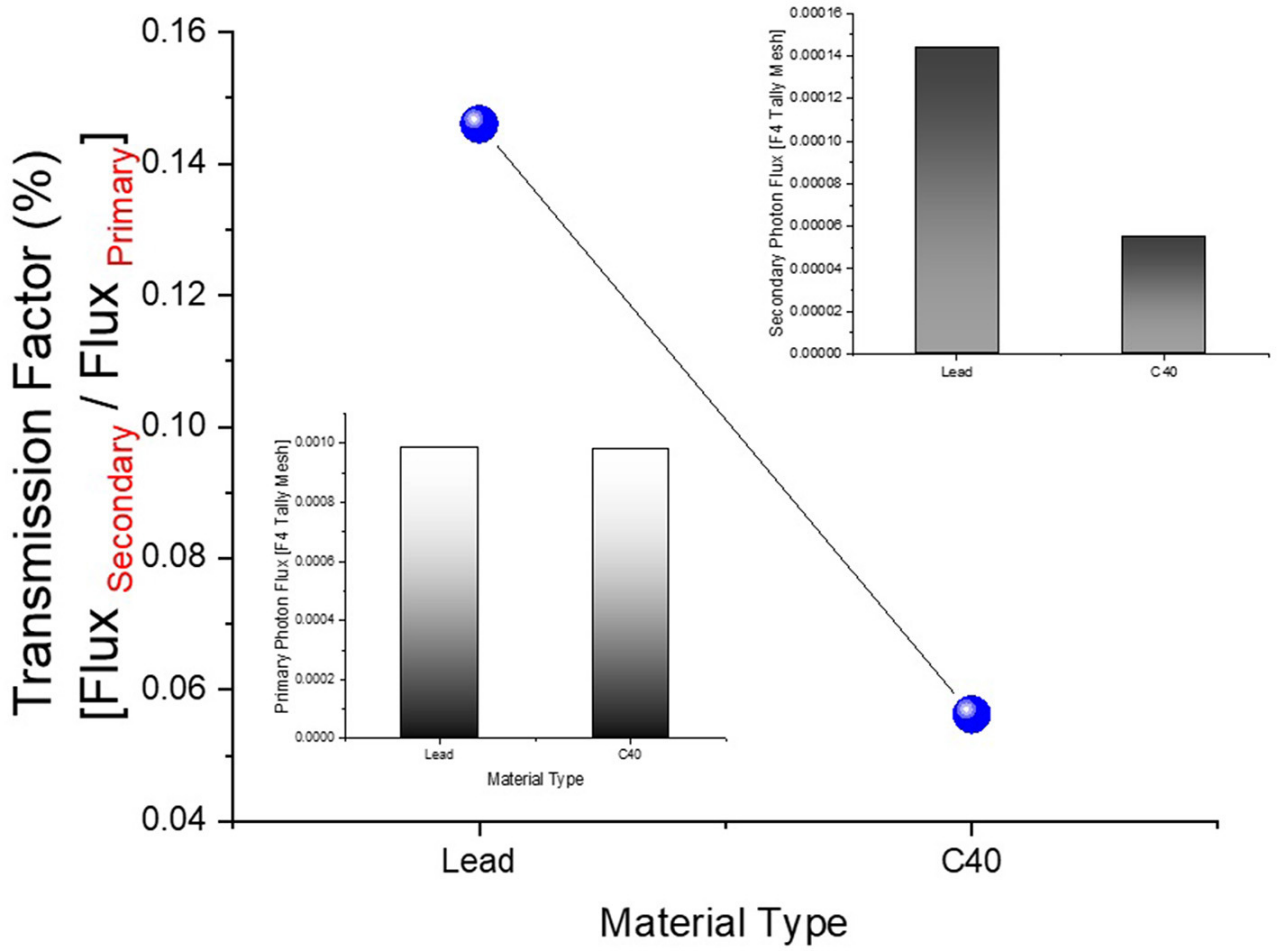
Variation of TF values for Lead-Acrylic and C40 shielding screens.

**Figure 10:**
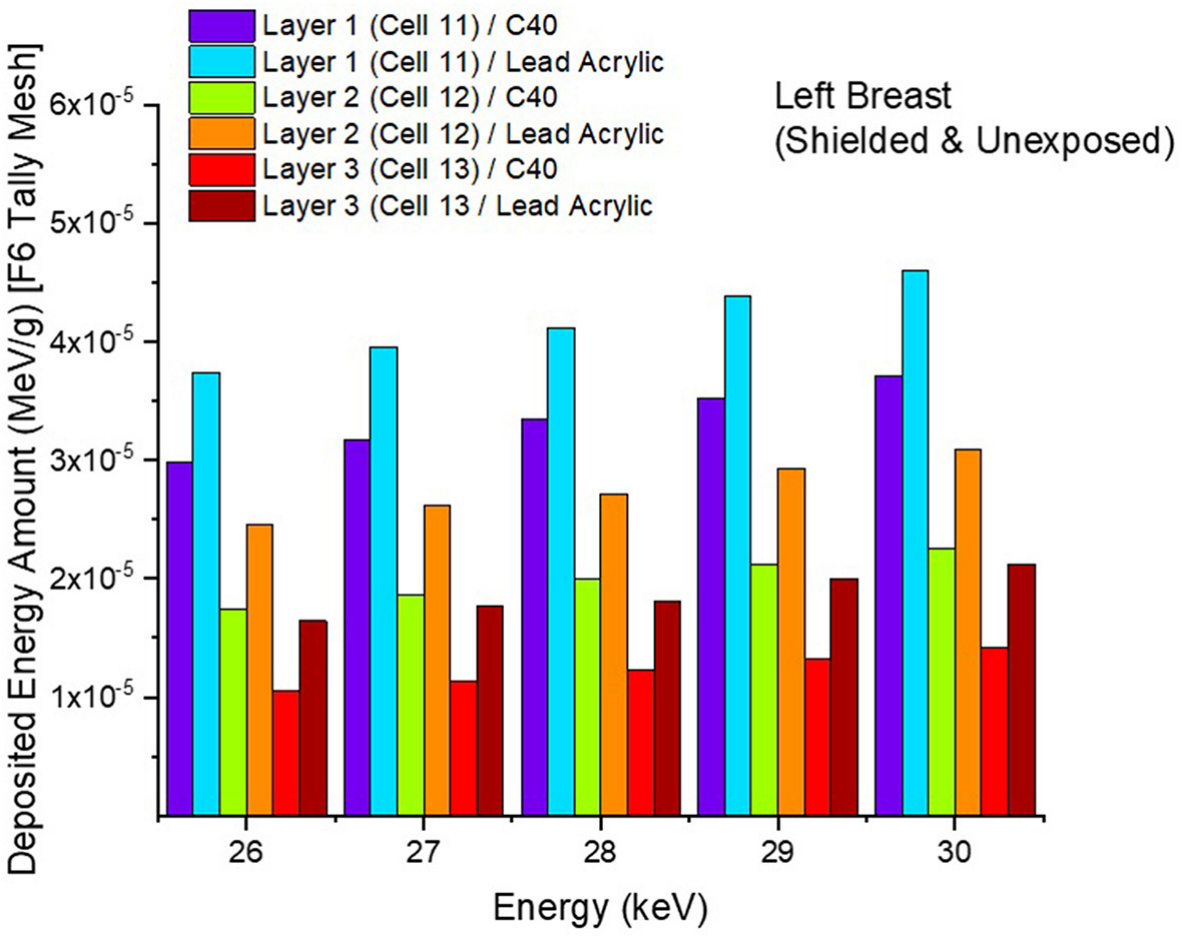
Variation of deposited energy amount in the three breast layers for unexposed and protected left breast.

**Figure 11:**
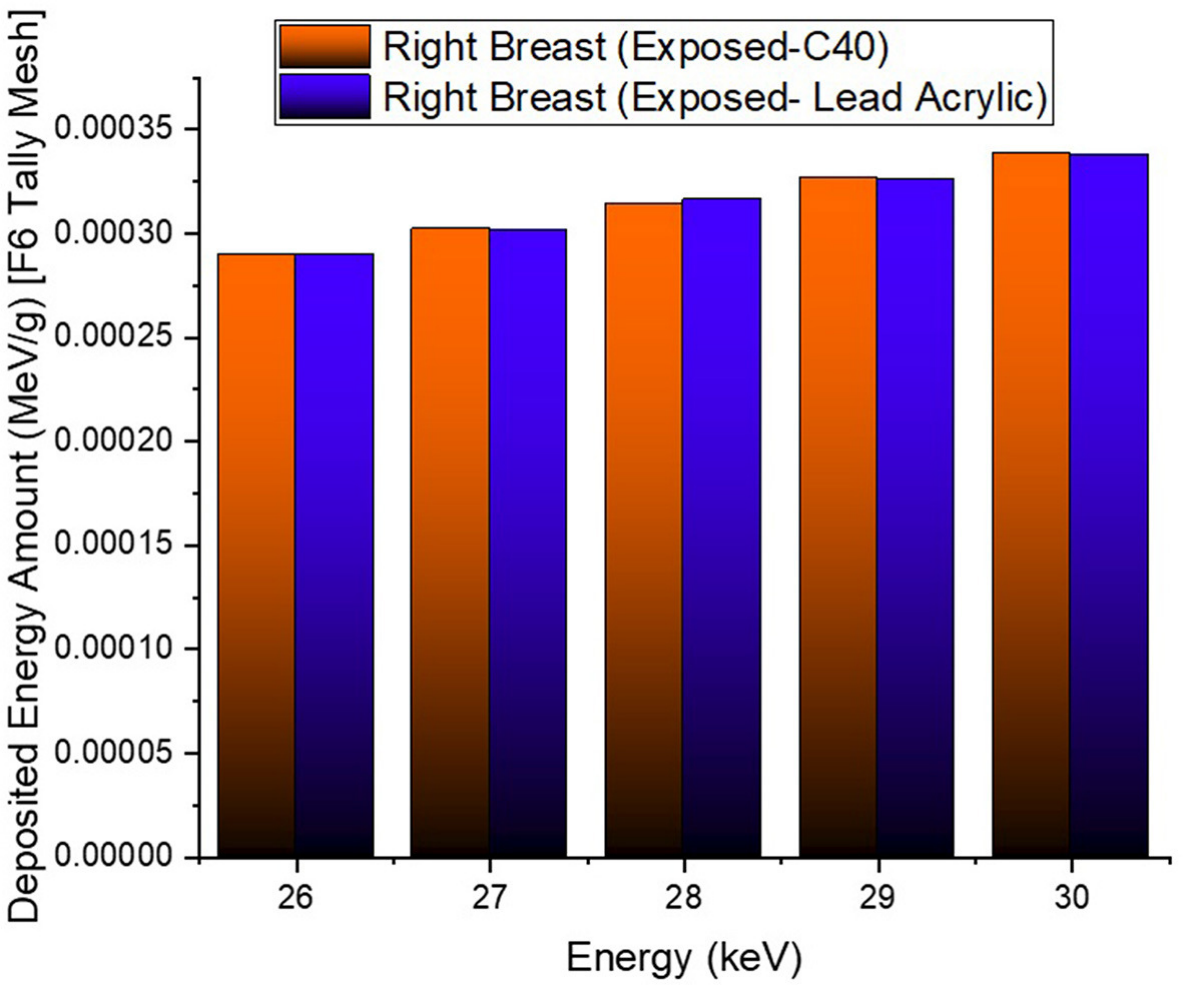
Variation of total deposited energy amount in the right breast.

**Figure 12:**
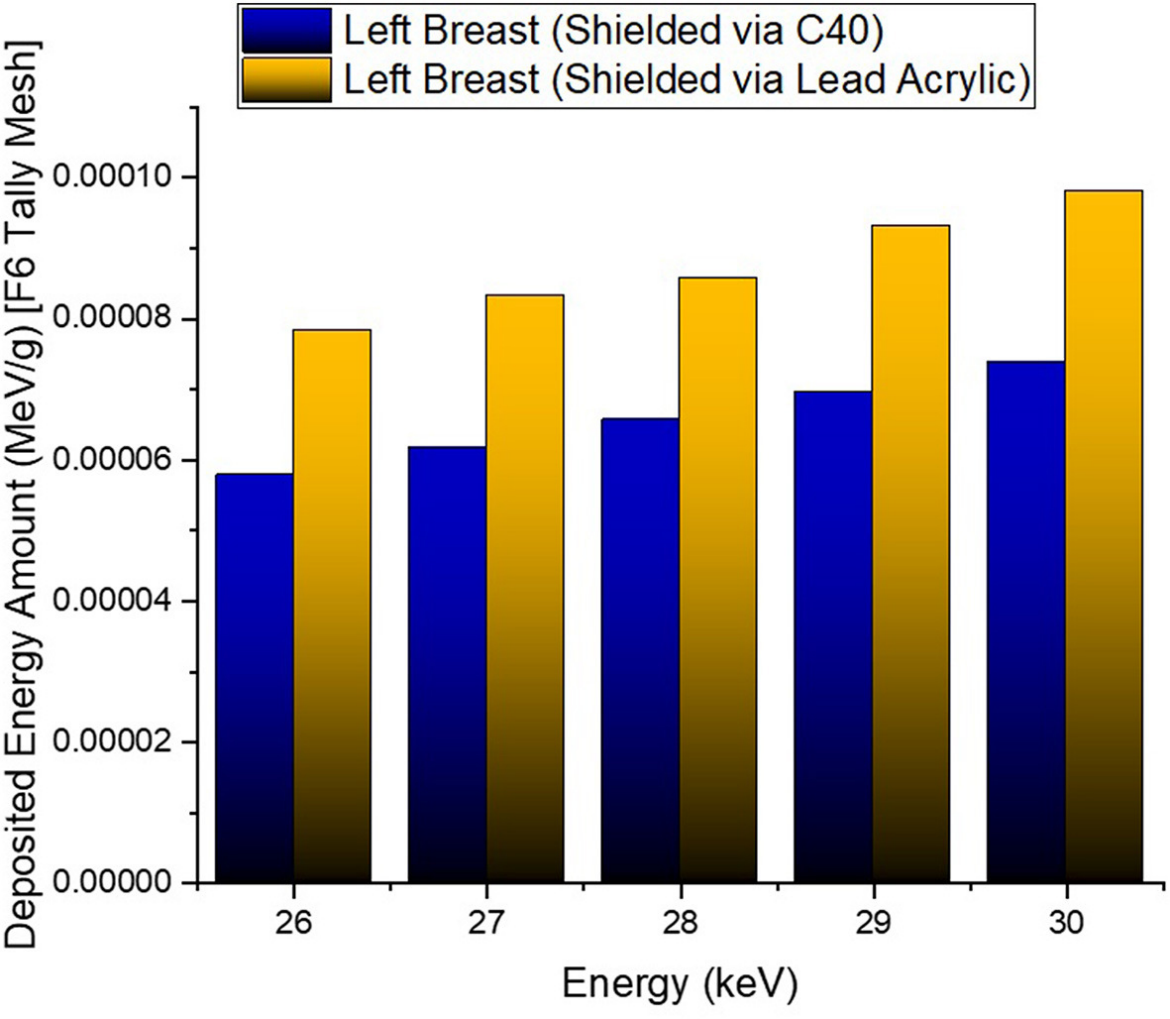
Variation of total deposited energy amount in the left breast.

In the second phase of the investigation, the total skin layer dose in the right and left breasts was determined for instances involving the use of lead acrylic and C40. Figure 1B depicts the 2 mm skin layer surrounding the breast as seen from the side. As shown in Figure 1B, cells 9, 10, and 11 represent independently modeled skin layers, and the output values for these cells were acquired and summed separately. This sum was taken into consideration as the overall skin dose per irradiation. Figure 8 shows the variations in the total skin dose of the right and left breasts when lead acrylic and C40 were utilized. As shown in Figure 9, the dose of the right breast did not vary for each energy value in either material. However, owing to the increase in energy, the ratio of total skin dosage in the right breast increased. For a given energy value, the dose to the unexposed left breast varied according to the use cases of lead acrylic and C40. If the unexposed left breast was shielded by a C40 protective monitor, the total skin dose calculated by three different cells for the left breast was far less for each energy value. This demonstrates that the quantity of dose received by the whole skin layer around the breast will be reduced when a C40 protective monitor is used as compared to a lead-acrylic monitor. Previous research (23) has shown that the lead-acrylic protective screen decreases the skin dose. This condition demonstrates that if the unexposed breast is shielded by an absorbent material, the skin dose, which also affects the glandular dose, would decrease, and this is seen as a crucial step in protecting the tissues around the examination area. This study's primary purpose was to demonstrate the potential differences between the transparent C40 protective glass screen and the lead-acrylic protective screen. In accordance with the objective, it has been found, based on TF and total skin dose values, that the C40 protective glass screen has superior protection properties compared to the lead-acrylic material and may be utilized to further reduce the total skin dose of the unexposed breast during the routine mammography examinations. In the third phase of the study, following the total skin dose reduction, the absorbed dose amounts for three different breast phantom layers were independently evaluated and compared. Figure 10 depicts the unexposed and shielded left breast dose as a function of increasing energy for the three modeled layers. As seen in the figure, the maximum dose is counted in the first layer for both lead acrylic and C40. The reason for this could be explained by the proximity of these three breast model layers to the X-ray source. As shown in Figure 3, layer 1 is the uppermost layer and the area that is geometrically closest to the X-ray source. In accordance with the ALARA principle, as the distance from the source increases, the exposure rate decreases in accordance with the inverse square law. This is also obvious from the difference between the total dose received by the first and second breast layers. The second and third breast layers, which are further away from the X-ray source than the first, were subjected to a lower dose. This is a significant result that may imply that greater emphasis should be placed on locations closer to the X-ray source during the protection of the unexposed breast during mammography. When C40 was utilized, however, the absorbed dose amounts in each of the three layers were seen to be lower. This is accurate for all three layers and is a key signal showing the overall superiority of C40 in protecting the breast that is not exposed and should be protected. Using the overall dose amounts as a sum of the three layers, the results for the three layers have been verified. Figures 11, 12 show the fluctuation of the total dose absorbed in the right and left breasts as a function of energy when lead acrylic and C40 are used as protective screens. As seen in Figure 11, the total dose received by the exposed right breast increased as the energy level increased. While dose ratios for the right breast did not have an important impact in the study, the purpose of this outcome is to emphasize the importance of using X-rays in the mammography technique and to illustrate that the protective screen used has no impact on the exposed breast. The minor differences seen in Figure 11 are attributable to the technical variations inherent to the Monte Carlo simulation. Figure 12 depicts the deposited energy amount in the unexposed and shielded left breast as the sum of the measured doses in the three layers for different X-ray energies. Even though the dose values absorbed in different breast layers vary as a function of distance from the source, the total dose absorbed in the left breast for C40 was found to be much less than that for lead-based acrylic. This change is reported to lead to improved protective properties for C40, with a difference of 35–38% for all energy levels.

## 4. Conclusion

Mammography, an X-ray-based diagnostic method, is the most critical phase in breast cancer screening programs. This is the standard procedure for screening programs for women over a certain age. This procedure might result in routine dose exposure as part of the diagnostic process, but it provides considerable health advantages. The objective of this research was to assess the radiation protection properties of a CdO-added glass screen (C40) by introducing a unique dose reduction strategy for mammography operations. Considering a previous clinical investigation, the C40 screen was designed with the same geometric proportions. The acquired outcomes were compared to the lead-acrylic material that was evaluated in the reference experimental study. The results indicate that the C40 glass screen is 35–38% more protective than the lead-acrylic screen. Considering the drawbacks of lead-hazardous acrylic's Pb structure, it can be concluded that glasses rich in cadmium oxide may be employed in mammography procedures since they provide better radioprotection than lead-acrylic and significantly reduce the dose amount in the unexposed left breast. This decrease will not only reduce the risk of breast cancer from radiation exposure but may also reduce radiation-induced cancer types. It can be concluded that the C40 protective screen would contribute to clinical applications as a shielding material for unexposed breasts due to its transparency in the visible light spectrum, ease of manufacture, and outstanding properties as a glass material. Many women have a mammogram every year. Nonetheless, there is a significant need for healthcare systems to focus on delivering high-quality services, with a strong emphasis on the patient experience. Therefore, protecting the unexposed breast is a wonderful way of spreading awareness of the importance of preventing unnecessary radiation exposure while providing the required medical care. On the other hand, based on the findings from this investigation, it has been demonstrated once again that Monte Carlo simulation-based radiation transport processes are an essential tool for medical radiation research. Particularly in studies concentrating on concept design, the implementation of extensive simulation techniques at this stage may serve as a crucial motivation for material manufacturing and the industrial phase. This research only examined exposed and unexposed breast tissues. In future studies, the current phantom might be expanded and examined for organs that are likely to suffer harm due to mammography procedures, including the brain, thyroid, heart, and lungs.

## Data availability statement

The raw data supporting the conclusions of this article will be made available by the authors, without undue reservation.

## Author contributions

GA: writing, simulations, and investigation. WE: writing and investigation. ER: writing, investigation, and data curation. GK, EI, and DS: investigation and data curation. AE: funding acquisition, investigation, and data curation. HT: writing, simulations, investigation, and supervision. All authors contributed to the article and approved the submitted version.

## References

[1] dos Santos AF De Almeida DRQ Terra LF Baptista MS Labriola L. Photodynamic therapy in cancer treatment-an update review. J Cancer Metast Treat. (2019) 5:25. 10.20517/2394-4722.2018.8335884903

[2] Azamjah N Soltan-Zadeh Y Zayeri F. Global trend of breast cancer mortality rate: a 25-year study. Asian Pacific J Cancer Prevent. (2019) 20:2015. 10.31557/APJCP.2019.20.7.2015 31350959 PMC6745227

[3] Homan SG Yun S Bouras A Schmaltz C Gwanfogbe P Lucht J. Breast cancer population screening program results in early detection and reduced treatment and health care costs for Medicaid. J Public Health Manage Pract. (2021) 27:70–9. 10.1097/PHH.0000000000001041 31592983

[4] Duffy SW Tabár L Yen AMF Dean PB Smith RA Jonsson H. Mammography screening reduces rates of advanced and fatal breast cancers: Results in 549,091 women. Cancer. (2020) 126:2971–9. 10.1002/cncr.32859 32390151 PMC7318598

[5] Dibden A Offman J Duffy SW Gabe R. Worldwide review and meta-analysis of cohort studies measuring the effect of mammography screening programmes on incidence-based breast cancer mortality. Cancers. (2020) 12:976. 10.3390/cancers12040976 32326646 PMC7226343

[6] Katalinic A Eisemann N Kraywinkel K Noftz MR Hübner J. Breast cancer incidence and mortality before and after implementation of the German mammography screening program. Int J cancer. (2020) 147:709–18. 10.1002/ijc.32767 31675126

[7] Heyes GJ Mill AJ Charles MW. Mammography—Oncogenecity at low doses. J Radiol Protect. (2009) 29:123. 10.1088/0952-4746/29/2A/S08 19454801

[8] Bushong SC. Radiologic Science for Technologists E-Book: Physics, Biology, and Protection. St. Louis: Elsevier Health Sciences. (2020).

[9] Šalát D Nikodemová D Klepanec A Lehotská V Šalátová A. Diagnostic reference levels in screening mammography centers in Slovakia. Radiat Prot Dosimetry. (2022) 198:537–9. 10.1093/rpd/ncac095 36005968

[10] Borrego-Soto G Ortiz-López R Rojas-Martínez A. Ionizing radiation-induced DNA injury and damage detection in patients with breast cancer. Genet Mol Biol. (2015) 38:420–32. 10.1590/S1415-475738420150019 26692152 PMC4763322

[11] Golubicic I Borojevic N Pavlovic T. Risk factors for breast cancer is ionizing radiation among them. J Buon. (2008) 13:487–94. 19145669

[12] Brown M Covington MF. Comparative benefit-to–radiation risk ratio of molecular breast imaging, two-dimensional full-field digital mammography with and without tomosynthesis, and synthetic mammography with tomosynthesis. Radiol Imaging Cancer. (2019) 1:e190005. 10.1148/rycan.2019190005 33778669 PMC7983792

[13] Fishman MD Rehani MM. Monochromatic X-rays: The future of breast imaging. Eur J Radiol. (2021) 144:109961. 10.1016/j.ejrad.2021.109961 34562745

[14] Hendrick RE. Radiation doses and cancer risks from breast imaging studies. Radiology. (2010) 257:246–53. 10.1148/radiol.10100570 20736332

[15] Vañó E Miller DL Martin CJ Rehani MM Kang K Rosenstein M. ICRP publication 135: diagnostic reference levels in medical imaging. Ann ICRP. (2017) 46:1–144. 10.1177/0146645317717209 29065694

[16] Elshami W Abuzaid MM Tekin HO. Effectiveness of breast and eye shielding during cervical spine radiography: an experimental study. Risk Manag Healthc Policy. (2020) 13:697. 10.2147/RMHP.S257185 32636688 PMC7335279

[17] Elshami W Tekin HO Issa SA Abuzaid MM Zakaly HM Issa B. Impact of eye and breast shielding on organ doses during cervical spine radiography: design and validation of MIRD computational phantom. Front Public Health. (2021) 9:751577. 10.3389/fpubh.2021.751577 34746086 PMC8569301

[18] Ali RM England A Mercer C Tootell A Walton L Schaake W. Mathematical modelling of radiation-induced cancer risk from breast screening by mammography. Eur J Radiol. (2017) 96:98–103. 10.1016/j.ejrad.2017.10.003 29103483

[19] Ali RMKM. Risk of Radiation-Induced Cancer From Screening Mammography. United Kingdom: University of Salford. (2016).33498015

[20] Popli MB Teotia R Narang M Krishna H. Breast positioning during mammography: mistakes to be avoided. Breast Cancer. (2014) 8:17617. 10.4137/BCBCR.S17617 25125982 PMC4125373

[21] Ali RM England A Mercer CE Tootell AK Hogg P. Impact of contralateral breast shielding on the risk of developing radiation-induced cancer from full-field digital mammography screening. J Med Imag Radiat Sci. (2019) 50:331–6. 10.1016/j.jmir.2019.02.005 31176442

[22] Pyka M Eschle P Sommer C Weyland MS Kubik R Scheidegger S. Effect of thyroid shielding during mammography: measurements on phantom and patient as well as estimation with Monte Carlo simulation. Eur Radiol Exper. (2018) 2:1–10. 10.1186/s41747-018-0042-9 29984353 PMC6022527

[23] Koo BY Lee KS. Reduction of scattered radiation dose by X-ray shielding during mammography. Radiat Phys Chem. (2020) 177:109111. 10.1016/j.radphyschem.2020.1091117124444

[24] Ara A Usmani JA. Lead toxicity: a review. Interdiscip Toxicol. (2015) 8:55. 10.1515/intox-2015-0009 27486361 PMC4961898

[25] Tekin HO ALMisned G Rammah YS Ahmed EM Ali FT Baykal DS. Transmission factors, mechanical, and gamma ray attenuation properties of barium-phosphate-tungsten glasses: Incorporation impact of WO3. Optik. (2022) 267:169643. 10.1016/j.ijleo.2022.169643

[26] Tekin HO ALMisned G Rammah YS Susoy G Ali FT Baykal DS. The significant role of WO3 on high-dense BaO–P2O3 glasses: transmission factors and a comparative investigation using commercial and other types of shields. Appl Phys A. (2022) 128:1–11. 10.1007/s00339-022-05620-y

[27] Tekin HO Ali FT Almisned G Susoy G Issa SA Ene A. Multiple assessments on the gamma-ray protection properties of niobium-doped borotellurite glasses: A wide range investigation using Monte Carlo simulations. Sci Technol Nucl Install. (2022) 2022. 10.1155/2022/5890896

[28] Zakaly HM Tekin HO Rammah YS Issa SA Alomari AH Ali FT. Physical features of high-density barium–tungstate–phosphate (BTP) glasses: elastic moduli, and gamma transmission factors. Electronics. (2022) 11:4095. 10.3390/electronics11244095

[29] RSICC Computer Code Collection. MCNPX user's manual version 2.4.0. Monte Carlo N-Particle Transport Code System for Multiple and High Energy Applications (2002).

[30] Chang TY Lai KJ Tu CY Wu J. Three-layer heterogeneous mammographic phantoms for Monte Carlo simulation of normalized glandular dose coefficients in mammography. Sci Rep. (2020) 10:2234. 10.1038/s41598-020-59317-4 32042071 PMC7010737

[31] Sechopoulos I Ali ES Badal A Badano A Boone JM Kyprianou IS. Monte Carlo reference data sets for imaging research: Executive summary of the report of AAPM Research Committee Task Group 195. Med Phys. (2015) 42:5679–91. 10.1118/1.4928676 26429242

[32] Kilic GÖ Kavaz E Ilik ER ALMisned G Tekin HO. CdO-rich quaternary tellurite glasses for nuclear safety purposes: Synthesis and experimental gamma-ray and neutron radiation assessment of high-density and transparent samples. Optical Mater. (2022) 129:112512. 10.1016/j.optmat.2022.112512

